# Under inflammatory stimuli mesenteric mesothelial cells transdifferentiate into macrophages and produce pro-inflammatory cytokine IL-6

**DOI:** 10.1007/s00011-019-01247-7

**Published:** 2019-05-21

**Authors:** Sándor Katz, Viktória Zsiros, Anna L. Kiss

**Affiliations:** Department of Anatomy, Histology and Embryology, Semmelweis University, Budapest, Hungary

**Keywords:** Mesothel/macrophage transition, Inflammation, Inflammatory cytokines

## Abstract

**Objective:**

Inflammatory stimuli inducing epithelial-to-mesenchymal transition (EMT) can transdifferentiate mesenteric mesothelial cells into macrophages.

**Methods:**

Sprague Dawley rat mesenteric mesothelial cells were used as a model. 1 ml Freund adjuvant was injected into the peritoneal cavity of rat and GM-CSF treatment was used to induce inflammation. IL-10 and IL-6 expression were studied by immunocytochemistry and Western blot analysis both in vivo and in vitro*.*

**Results:**

Control mesothelial cell express anti-inflammatory IL-10, but no pro-inflammatory IL-6 expression could be detected in them. By the time of inflammation, IL-6 expression increased (reached the maximum level at the fifth day of inflammation), parallel to this the IL-10 entirely disappeared from these cells. In vitro GM-CSF treatment resulted in similar changes. As the mesothelial cells started to recover (at the eighth day of inflammation) IL-6 expression decreased and IL-10 level started to increase again.

**Conclusion:**

These data show that under inflammatory stimuli mesothelial cells—like macrophages—can produce pro-inflammatory cytokines.

## Introduction

Monocytes and macrophages play important role in immune responses. Under healthy conditions large number of these phagocytic cells reside in the peritoneal cavity as self-sustaining resident macrophages [1, 2]. During inflammation a heterogeneous population of phagocytes appear in the peritoneal cavity [3]. It seems likely that besides of activated tissue-resident, and infiltrating monocyte-derived macrophages, cells originating from non-hemopoietic sources should also contribute to this subset of macrophages.

Earlier we proved, that inflammation transdifferentiated mesenteric mesothelial cells into macrophages-like cells. During this transition mesothelial cells expressed ED1 (pan-macrophage marker), they intensively phagocytosed ink- and bio-particles [4]. Similar changes could be observed when primary mesenteric cultures were treated with granulocyte–macrophage colony-stimulating factor (GM-CSF), which promotes the survival and activation of granulocytes, macrophages as well as dendritic cell differentiation in vivo, stimulates proliferation of several non-hemopoietic cell types [5]. In recent work, we followed the expression of pro- (IL-6) and anti-inflammatory cytokines (IL-10) in mesenteric mesothelial cells.

## Materials and methods

1 ml complete Freund’s adjuvant (Sigma, Saint Louis, Missouri) was injected into the peritoneal cavity of male rats (200–250 g). Mesentery was isolated from control and treated animals (10 animals per group). For in vitro experiments, mesentery was cut out from control animals, maintained in Dulbecco’s Modified Eagle Medium (Nutrient Mixture F-12 (DMEM/F12, Life Technologies, Paisley, UK) and were treated with 1 ng/ml GM-CSF (Sigma, Saint Louis, Missouri) for 3 days. For immunolabeling, we applied a modified Tokuyashu technique [6]. The 0.6-µm-thick frozen sections were cut by Leica Ultracut S ultramicrotome (Vienna, Austria).

*For immunocytochemistry*, anti-IL-6 (1:100 ABCAM, Cambridge, UK), and anti-IL-10 (1:500 ABCAM, Cambridge, UK) were used as primary antibodies, anti-mouse and anti-rabbit IgG Alexa Fluor 488 (1:200, Molecular Probes, Leiden, The Netherlands) were applied as secondary antibodies. The nuclei were stained with DAPI (Vector Laboratories Inc. Burlingame, California). The samples were observed with Zeiss LSM 780 confocal microscope. Images were performed by Photoshop Elements 15 Editor.

### Western blot analysis

The Western blot analysis was performed as it was previously described [4, 7]. Band densities were measured by the ImageJ software (U.S. National Institutional of Health, Bethesda, Maryland) and analyzed by the Microsoft Excel 2013 program.

## Results

**Fig. 1:**
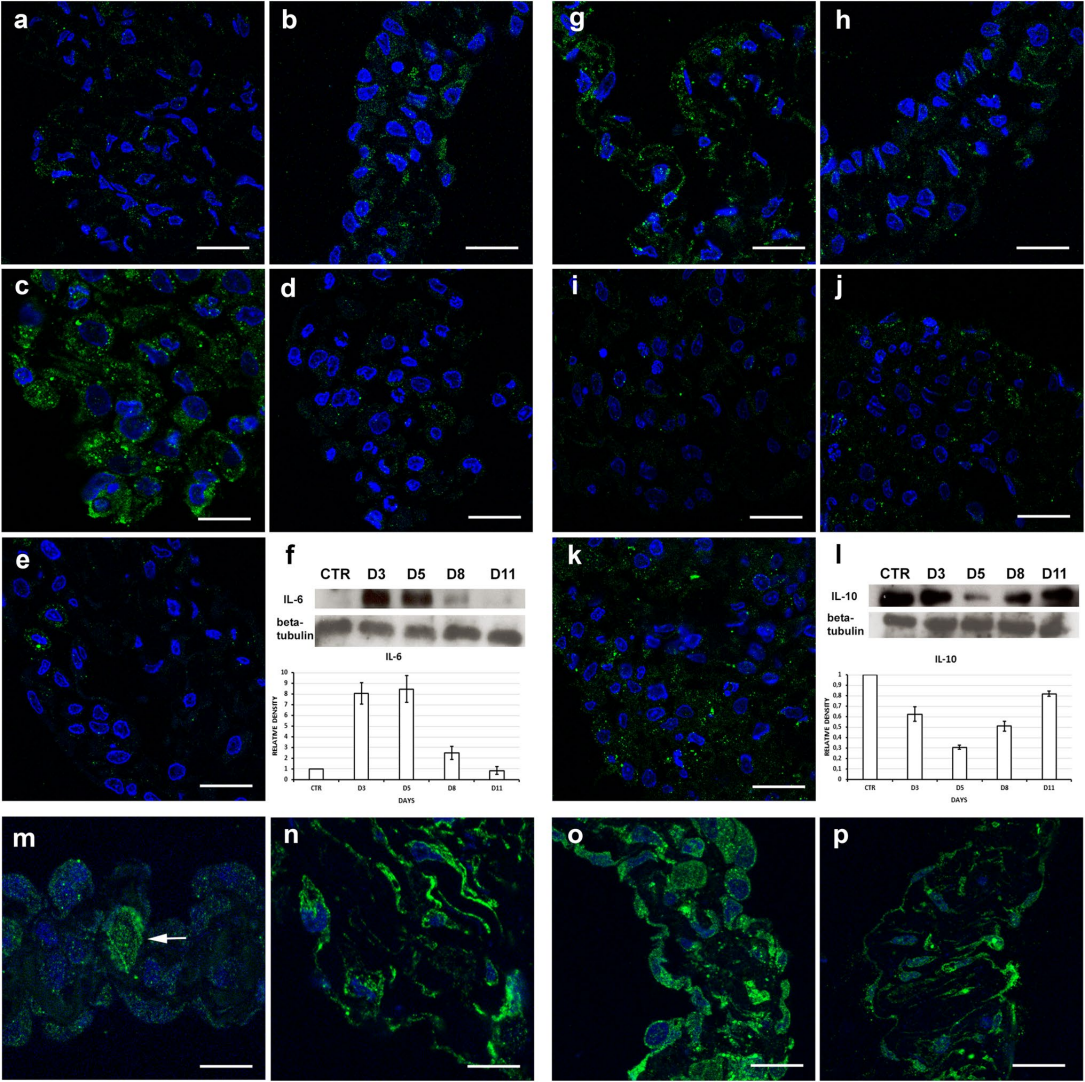
**a**–**f** IL-6 expression in vivo. **a** Control mesothelial cells do not express IL-6; a significant level of: IL-6 was expressed at the 3rd day (**b**), reaching the highest level at the 5th day (**c**); and decreased markedly at the 8th day (**d**); entirely diminished at the 11th day, when the regeneration finished (**e)**. **f** Western blot results and densitometry show the same tendency. **g**–**l** IL-10 expression in vivo. **g** Control mesothelial cells express significant amount of anti-inflammatory IL-10. **h**–**j** At the 3rd, 5th and 8th days of inflammation, the IL-10 expression is gradually and significantly decreased; **k** at the 11th day, significant amount of IL-10 could be detected again in the mesothelial cells. **l** These results are supported by Western blot results and densitometry. **m**, **n** IL-6 expression in vitro. **m** Control cells do not express significant amount of IL-6; **n** 3 days GM-CSF treatment resulted in an intensive IL-6 expression. **o**, **p** IL-10 expression in vitro. **o** IL-10 is strongly expressed in control cells; **p** 3 days GM-CSF treatment significantly decreased the expression of IL-10. Bars **a**, **b** 15 µm; **c** 12 µm; **d**, **e**, **g**–**k** 15 µm; **m**: 12 µm; **n**–**p** 13 µm

Control mesothelial cell expresses anti-inflammatory IL-10 (Fig. 1g), but no pro-inflammatory IL-6 expression could be detected in them (Fig. 1a). At the third day of inflammation, the mesothelial cells started to express IL-6 (Fig. 1b), reached the maximum level at the peak time of inflammation (fifth day; Fig. 1c). As the mesothelial cells started to recover (at the 8th day of inflammation), IL-6 expression decreased (Fig. 1d) and practically no IL-6 expression was detected in mesothelial cells at 11th day (Fig. 1e). In contrast to this, no IL-10 could be detected at the peak time of inflammation (Fig. 1i), but its level increased again after the eighth day of inflammation. (Fig. 1j). Our Western blot results supported these immunocytochemical observations (Fig. 1f, l). In vitro GM-CSF treatment resulted in similar changes in mesothelial cells (Fig. 1m–p).

## Discussion

Monocytes and macrophages are the main sources of the inflammatory cytokines, but activated lymphocytes, fibroblasts and endothelial cells can also produce them [8]. To provide additional data about mesothelial cells/macrophage transition, we investigated the expression of IL-6 and IL-10. Our recent results show that healthy mesenteric mesothelial cells express IL-10, the main suppressor of macrophage activation. No IL-6 expression could be detected in them. Under inflammatory stimuli and GM-CSF treatment, IL-10 expression significantly diminished in these cells, and they started to produce IL-6, indicating that inflammation and GM-CSF treatment induced macrophage-like phenotypic change in them. Macrophages are the major producers of tumor necrosis factor, TNFα as well [9]. We also proved that inflammation induced TNFα expression in mesenteric mesothelial cells reaching the highest level at the peak time of inflammation [4]. Control mesothelial cells did not express EGR1, one of the main regulator of TNFα production, and monocyte–macrophage differentiation [10–12], but by the time of inflammation EGR1 expression increased significantly in them and it was translocated into the nuclei of these cells [4]. All these data strongly support the idea that under inflammatory stimuli mesothelial cells can transdifferentiate into macrophages.

## Conclusion

Hereby, we provide further evidence about transdifferentiation of mesothelial cells to macrophages. These transformed mesothelial cells largely contribute to macrophage population, appearing in large number in the peritoneal cavity during inflammation.
